# Global, Regional, and National Burden of Burn Injury by Total Body Surface Area (TBSA) Involvement from 1990 to 2021, with Projections of Prevalence to 2050

**DOI:** 10.3390/healthcare13162077

**Published:** 2025-08-21

**Authors:** Nara Lee, Youngoh Bae, Suho Jang, Dong Won Lee, Seung Won Lee

**Affiliations:** 1 Department of Plastic and Reconstructive Surgery, Yonsei University College of Medicine, Seoul 03722, Republic of Korea; 2 Institute for Human Tissue Restoration, Severance Hospital, Yonsei University College of Medicine, Seoul 03722, Republic of Korea; 3 Department of Precision Medicine, Sungkyunkwan University School of Medicine, Suwon 16419, Republic of Korea; 4 Department of Medical AI, Sungkyunkwan University School of Medicine, Suwon 16419, Republic of Korea; 5 Department of Artificial Intelligence, Sungkyunkwan University, Suwon 16419, Republic of Korea; 6 Department of Metabiohealth, Sungkyunkwan University, Suwon 16419, Republic of Korea; 7 Personalized Cancer Immunotherapy Research Center, Sungkyunkwan University School of Medicine, Suwon 16419, Republic of Korea; 8 Department of Family Medicine, Kangbuk Samsung Hospital, Sungkyunkwan University School of Medicine, Seoul 03181, Republic of Korea

**Keywords:** burn injury, total body surface area, global burden of disease, prevalence, health status disparities, socioeconomic factors, global health

## Abstract

Background/Objectives: Burn injuries are a major public health concern. This study estimated global, regional, and national burn burdens by total body surface area from 1990 to 2021 and projected trends to 2050. Methods: Utilizing data from the Global Burden of Disease Study 2021, we examined the prevalence, mortality, and years lived with disability (YLDs) according to age, sex, and region. Future trends were predicted using Bayesian meta-regression models and Das Gupta decomposition analysis. Results: In 2021, global prevalence was 12.99 million for severe burns and 235.34 million for mild burns, with age-standardized rates of 158.75 and 2815.26 per 100,000. Severe burns were highest in Southern Latin America (7836.51 per 100,000) and mild burns in the Caribbean (626.94 per 100,000). The largest declines from 1990 to 2021 were in high-income North America for severe burns (−38.22%) and East Asia for mild burns (−73.03%). Females had higher severe burn prevalence at younger and older ages, while males had higher mild burn prevalence from early adulthood. Leading risk factors were fire, heat, and hot substances (38.22% of severe burn YLDs; 53.87% for mild burns). By 2050, severe burns are projected to rise by 233.4% and mild burns by 142.5%, with Eastern Europe showing the largest growth. Conclusions: Although age-standardized burn rates are declining, absolute cases are projected to rise due to population growth and aging, particularly in low- and middle-income countries, underscoring the need for stronger prevention and improved burn care infrastructure.

## 1. Introduction

Burn injury refers to traumatic damage to the skin and deep tissues caused by external factors such as heat, chemicals, electricity, and radiation [1]. The severity of burn injuries is classified based on the percentage of total body surface area (TBSA) affected. Burn injuries involving less than 20% TBSA are generally considered mild, whereas those affecting 20% or more are classified as severe. Severe burn injuries carry a substantially higher risk of mortality and complications [2]. Patients with severe burns frequently require prolonged hospitalization and intensive care owing to extensive tissue damage, along with an increased risk of complications such as infections, sepsis, and multiple organ failure [3]. Additionally, survivors of burn injuries frequently need ongoing skin grafting procedures, physical therapy, and psychological rehabilitation [4].

Burn injuries impose a substantial socioeconomic burden [5]. Treating severe burns demands considerable medical resources, including surgical interventions, intensive care unit management, and long-term rehabilitation [3,6]. The low rate of return to work and associated loss of productivity further amplify the economic impact [7]. In low- and middle-income countries (LMICs), the scarcity of specialized burn treatment facilities and trained medical personnel markedly reduces survival rates and recovery outcomes [8]. Moreover, survivors of burn injuries encounter an increased risk of long-term physical disabilities and psychological conditions, such as post-traumatic stress disorder, depression, and social isolation. These challenges impact not only the individuals but also their families and communities [9].

The Global Burden of Disease (GBD) study provides a detailed framework for evaluating the global health impact of burn injuries. By analyzing years lived with disability (YLDs) and using age-standardized rates, the GBD data facilitate direct comparisons between burn injuries and other notable health issues. However, despite growing recognition of burn injuries as a public health issue, high-quality, country-specific data on their prevalence and long-term outcomes are still limited.

Furthermore, projecting the prevalence of burn injuries is crucial for preparing for future healthcare needs, especially in areas experiencing rapid urbanization, population aging, and high rates of household and occupational injuries. Accurately assessing the burden of severe burns is vital for refining public health policies and optimizing resource distribution. These projections aid in establishing priorities for policy interventions and improving the efficacy of prevention strategies. Over time, strategic allocation of resources can enhance efforts to prevent burn injuries and improve patient outcomes.

In this study, we utilized GBD data from 1990 to 2021 to analyze global, regional, and national burn prevalence, mortality, and YLDs of burn injuries by age, sex, and location. By integrating demographic shifts and socioeconomic trends, we projected TBSA-based burn injury prevalence up to 2050, providing a robust, data-driven foundation for healthcare policy development and resource planning. The results deliver crucial insights for tailoring prevention and treatment strategies across diverse healthcare settings.

## 2. Materials and Methods

### 2.1. Study Overview

This study employed data from the GBD 2021 to estimate global prevalence, mortality, and YLDs related to burns, stratified by age, sex, and year. We extended future prevalence projections to 2050 using predictive modeling. The research followed the GBD standard protocol and adhered to the Guidelines for Accurate and Transparent Health Estimates Reporting (GATHER) [10].

### 2.2. Case Definition

Burn injuries were defined according to the GBD criteria, encompassing trauma caused by heat, chemicals, electricity, or radiation that results in damage to the skin and underlying tissues. In line with the GBD 2021 methodology, burn severity was classified based solely on TBSA involvement, given the absence of standardized, globally available depth-specific data. Specifically, burns involving <20% TBSA were categorized as mild, and those involving ≥20% TBSA were categorized as severe, and this classification was applied uniformly across all age groups in this study.

Only cases resulting in physical or functional impairment lasting at least 24 h were included, consistent with the GBD definition for estimating YLDs DALYs. All data were harmonized and standardized using GBD data processing protocols.

### 2.3. Data Sources

This study utilized the GBD 2021 Data Input Sources, which are publicly available via the Global Health Data Exchange (GHDx) platform (https://ghdx.healthdata.org/gbd-2021/data-input-sources, accessed on 10 February 2025). Estimates of burn injury prevalence and YLDs from 1990 to 2021 were derived from various sources, including population-based studies, national health surveys, hospital and emergency medical records, global health surveillance systems, and collaborative research contributions.

The dataset included cases of thermal, chemical, and electrical burn injuries, categorized according to GBD case definitions. Additionally, data on key risk factors such as unintentional injuries, fire, heat and hot substances, self-harm and interpersonal violence, and transport-related injuries were incorporated. Each dataset was assigned a unique identifier and registered within GHDx, with final estimates generated in accordance with GBD data validation and quality assessment protocols.

### 2.4. Modeling and Data Processing

A Bayesian meta-regression analysis was conducted using the rstanarm package in R to estimate burn injury prevalence and YLDs across age, sex, region, and year. Bayesian inference, implemented via rstanarm, ensured robust parameter estimation [11,12].

### 2.5. Data Preprocessing

Before modeling, data preprocessing involved the following steps: (i) separating datasets that covered broad age groups or lacked sex-specific data; (ii) applying proportional allocation methods to address missing demographic information; and (iii) harmonizing case definitions using network meta-regression in accordance with GBD standards. Adjustments for sex-specific data and age distributions followed established GBD methodological frameworks [13]. For datasets without sex-specific estimates, the Bayesian meta-regression tool MR-BRT (meta-regression–Bayesian, regularized, trimmed) was used to estimate and normalize sex ratios [14,15].

Alternative definitions of burn injuries were standardized to align with GBD case definitions. Bias correction was applied using MR-BRT, with adjustments based on the logit difference in prevalence across various case definitions. Outlier detection was performed by identifying values that exceeded 1.5 times the median absolute deviation of age-standardized prevalence rates.

Prevalence estimates were generated using DisMod-MR 2.1, a Bayesian hierarchical model for disease burden estimation that incorporates age-, sex-, region-, and year-specific covariates. For children under five, burn-related injuries were assumed to be rare, and appropriate adjustments were made. Uncertainty intervals (UIs) were calculated by acquiring the 2.5th and 97.5th percentiles from 1000 posterior draws.

To compute YLDs, age-, sex-, region-, and year-specific disability weights were applied to prevalence estimates, with adjustments made for comorbid conditions. Prevalence and burden rates are presented at the global, regional (7 super-regions and 21 sub-regions), and national levels.

The GBD framework quantifies the impact of various diseases and injuries, primarily focusing on direct causes of mortality. While some burn cases directly result in death, several contribute to mortality through secondary complications. In such instances, the complication—rather than the burn itself—is recorded as the primary cause of death.

The final model used the following equation:

(1) $$Logit\;\left(predicted\;prevalence\right)=\;\beta_{1}\left(SDI\right)+\;\alpha_{l,a,s}$$

where *β*_1_ represents a fixed coefficient for the socio-demographic index (SDI), and *α* represents random intercepts for location (*l*), age group (*a*), and sex (*s*). The projected number of cases was computed by multiplying the predicted prevalence by population estimates. To adjust for temporal variations, the 2020 prevalence estimate from GBD 2021 was used as a baseline, with adjustments applied to all projections extending to 2050.

### 2.6. Risk Factor Estimation

This study assessed various risk factors contributing to burn incidence, including unintentional injuries, fire, heat and hot substances, self-harm and interpersonal violence, and transport-related injuries. To ensure accuracy, risk factor estimates were adjusted using GBD parameter-based meta-regression models, which accounted for potential confounders. Relative risk estimates were derived from representative population data, while summary exposure values (SEVs) were used to quantify the contribution of each risk factor to the overall burn burden. By integrating risk factor distribution and exposure levels, SEVs provided a comprehensive assessment of their impact on burn prevalence.

### 2.7. Future Projections

To forecast burn prevalence through 2050, a regression model incorporating the SDI and age–sex stratification was applied. This model accounted for SDI and temporal variations to estimate future prevalence rates. The projected prevalence was then multiplied by future population estimates to determine the expected total number of burn cases.

Additionally, a Das Gupta decomposition analysis was conducted to quantify the relative contributions of population growth, aging, and non-demographic prevalence trends to the projected increase in burn cases between 2021 and 2050 [16]. Model validation was performed using backward projection testing, in which prevalence estimates from 1990 to 2020 were used to forecast prevalence from 2030 to 2050. These projections informed YLD-based disability burden calculations.

## 3. Results

### 3.1. Global Prevalence of Burns in 2021

The global prevalence of burn injury cases in 2021 was estimated at 12.99 million (95% UI: 11.96–14.29 million) for severe burns and 235.34 million (95% UI: 214.09–259.39 million) for mild burns (Table 1). The corresponding age-standardized prevalence rates were 158.75 (95% UI: 146.05–174.66) per 100,000 for severe burns and 2815.26 (95% UI: 2565.28–3104.57) per 100,000 for mild burns, indicating a 17.7-fold higher burden of mild burns when adjusted for population structure.

From 1990 to 2021, the global age-standardized prevalence rate of severe burns decreased by 0.54% (95% UI: −0.56 to −0.51), while mild burns declined by 0.31% (95% UI: −0.32 to −0.29). Most GBD regions showed declining trends in both categories, with the steepest reductions observed in East Asia, Central Latin America, and Andean Latin America. However, Oceania and the Caribbean exhibited modest increases in prevalence, particularly among adults.

Although age-standardized burn rates are declining, the absolute number of cases is projected to rise due to population growth and aging—particularly in low- and middle-income countries. Moreover, the vast number of mild burn cases among children and adolescents highlights the persistent burden in younger populations and the importance of targeted, age-specific prevention efforts.

### 3.2. Regional Prevalence of Burns

**Figure 1:**
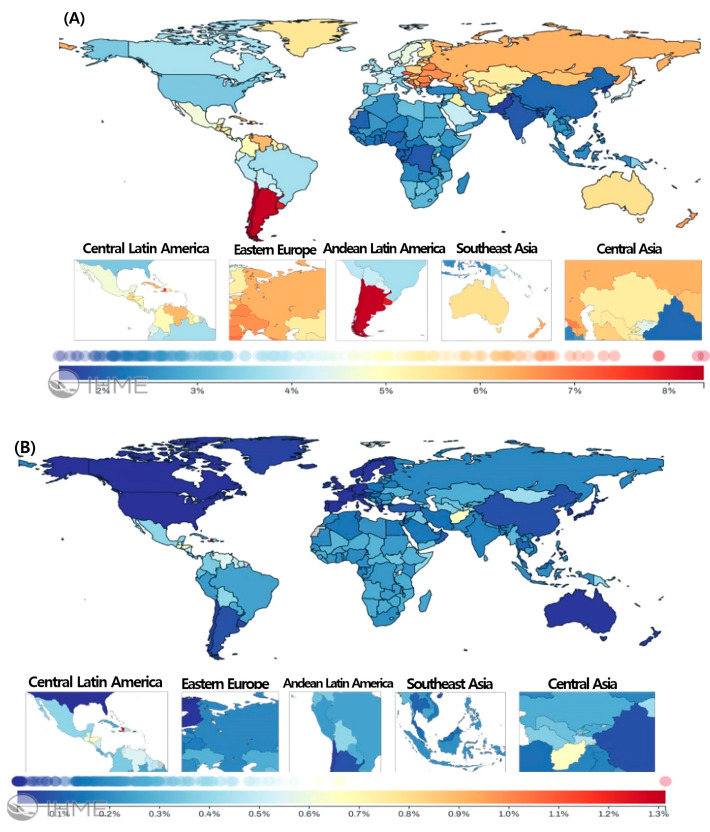
Age-standardized prevalence of burn injury involving <20% (**A**) and ≥20% (**B**) total body surface area (TBSA) by country for male and female sexes combined and all ages in 2021.

In 2021, the regional distribution of burn injury prevalence demonstrated marked variation across the 24 GBD regions (Table 1, Figure 1). For severe burns, the highest prevalence was noted in the Caribbean, reaching 626.94 per 100,000 (95% UI: 516.55–802.54), followed by Central Latin America (407.23 per 100,000, 95% UI: 365.35–456.89). The lowest prevalence rates were observed in high-income North America (13.99 per 100,000, 95% UI: 12.91–15.12) and Western Europe (16.01 per 100,000, 95% UI: 14.76–17.46). The steepest declines in mild burn prevalence during the same period were found in East Asia (−73.03% [95% UI: −73.01% to −72.87%]) and Central Europe (−69.16% [95% UI: −69.16% to −68.85%]).

For mild burns, the highest age-standardized prevalence was observed in Southern Latin America, at 7836.51 per 100,000 population (95% UI: 6939.49–8972.91), followed by Central Europe (5996.47 per 100,000, 95% UI: 5467.77–6655.23). In contrast, the lowest prevalence rates were recorded in South Asia (1844.24 per 100,000, 95% UI: 1665.64–2026.5) and East Asia (1935.22 per 100,000, 95% UI: 1750.03–2142.56). Between 1990 and 2021, the most substantial relative declines in severe burn prevalence occurred in high-income North America (−38.22% [95% UI: −38.33% to −38.44%]) and Tropical Latin America (−37.31% [95% UI: −37.19% to −37.66%]).

The age-standardized prevalence of severe burns in the Caribbean was approximately 47.5 times higher than in high-income North America, whereas the difference in mild burns between Southern Latin America and South Asia was about 2.6-fold. This disparity was more pronounced for severe burns. These variations likely reflect not only true differences in incidence but also inconsistencies in case reporting, diagnostic criteria, and data quality across countries. Additionally, broader structural factors—such as socioeconomic status, housing safety, burn prevention infrastructure, and access to trauma care—may play a significant role. High-income regions consistently showed the lowest burden, while several Latin American and Eastern European regions experienced disproportionately high prevalence, particularly among children and adolescents.

### 3.3. National Prevalence of Burns

In 2021, the highest age-standardized prevalences of severe burns were observed in Guatemala (1211.72 per 100,000) and El Salvador (1201.61 per 100,000), while the lowest were in the Netherlands (15.7 per 100,000) and San Marino (16.56 per 100,000). Between 1990 and 2021, the largest relative reductions occurred in Estonia (−77.52%) and the Republic of Korea (−77.03%), with YLDs declining most in the Republic of Korea (−85.53%) and China (−85.32%).

For mild burns, Argentina (7875.42 per 100,000) and Chile (7844.03 per 100,000) showed the highest prevalence, whereas Taiwan (Province of China) (1419.99 per 100,000) and the Democratic People’s Republic of Korea (1470.36 per 100,000) recorded the lowest. The greatest declines in prevalence were observed in Mauritius (−49.48%) and Taiwan (−46.78%), with the largest YLD reductions also occurring in Mauritius (−49.54%) and Taiwan (Province of China) (−47.07%).

These patterns reflect underlying disparities in burn prevention infrastructure, healthcare access, and socioeconomic vulnerability, particularly in regions undergoing rapid urbanization or lacking specialized burn care systems.

### 3.4. Prevalence by Sex and Age

**Figure 2:**
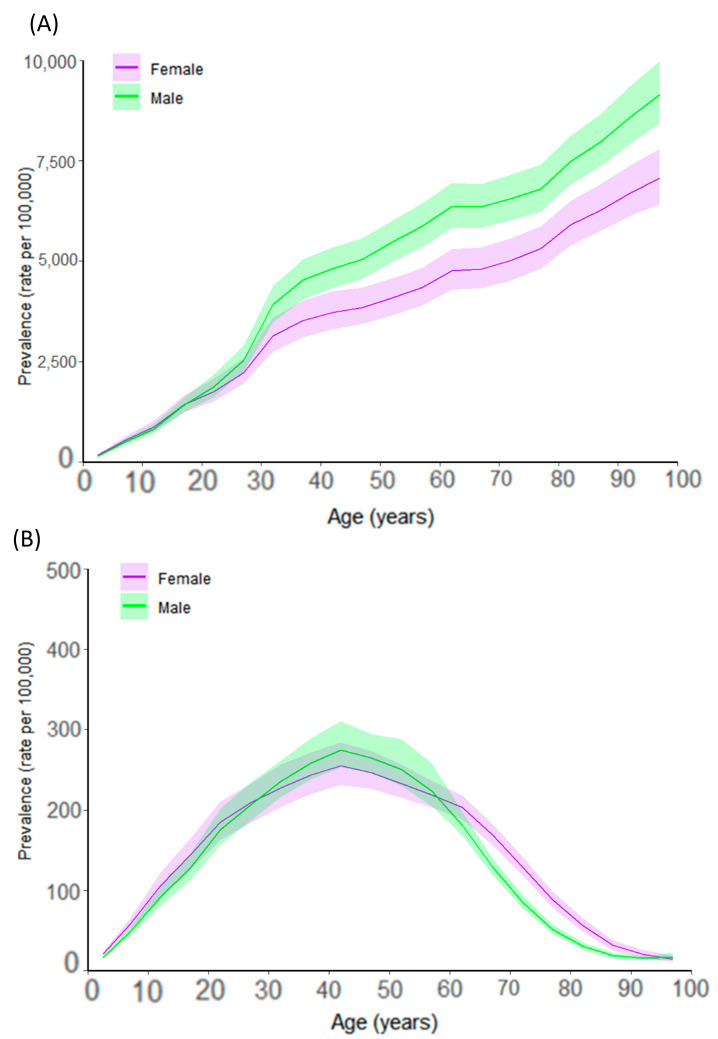
Global prevalence of burn injury involving <20% (**A**) and ≥20% (**B**) total body surface area (TBSA) by age and sex in 2021. Shaded areas represent 95% uncertainty intervals.

Globally, females had a higher age-standardized prevalence of severe burns (142.93 per 100,000; 95% UI: 102.33–183.53) than males (135.11 per 100,000; 95% UI: 89.89–180.33). Females exhibited a higher prevalence up to age 19, although this difference diminished in the 20–29 age group. Males aged between 39 and 59 years had a higher prevalence, whereas the prevalence in females increased sharply from age 60 onward, particularly in the 65–79 age group. Males aged 95+ years had a slightly higher prevalence (Figure 2).

Regarding mild burns, males had a higher age-standardized prevalence (4810.89 per 100,000; 95% UI: 3514.04–6107.74) than females (3774.89 per 100,000; 95% UI: 2810.68–4739.09). While females were more vulnerable at younger ages, prevalence in males increased significantly from the 20s onward, with substantially higher rates observed in those aged 30 and older. The largest sex disparity was observed in those aged 90+ years.

Between 1990 and 2021, YLDs for both severe and mild burns declined across all age groups. Females consistently exhibited higher YLDs for severe burns, whereas males had higher YLDs for mild burns, a pattern that persisted across all age groups.

These patterns reflect age- and gender-specific vulnerabilities. Young females in low-resource settings are disproportionately affected due to early domestic responsibilities and exposure to unsafe cooking environments. In contrast, occupational hazards explain higher rates in adult males. Prevalence spikes again in the elderly, likely due to frailty and delayed response to hazards.

### 3.5. Key Risk Factors Associated with Burns in 2021

Severe burns were primarily attributed to fire, heat, and hot substances (38.22% of total YLDs; 95% UI: 24.13–54.21%), self-harm, and interpersonal violence (7.78%; 95% UI: 5.59–10.80%), and transport injuries (3.05%; 95% UI: 2.38–3.71%).

For mild burns, fire, heat, and hot substances accounted for 53.87% of YLDs (95% UI: 37.07–69.15%), followed by unintentional injuries (8.52%; 95% UI: 5.88–11.91%) and self-harm and interpersonal violence (2.80%; 95% UI: 1.72–4.11%).

### 3.6. Projected Burn Cases by 2050

**Figure 3:**
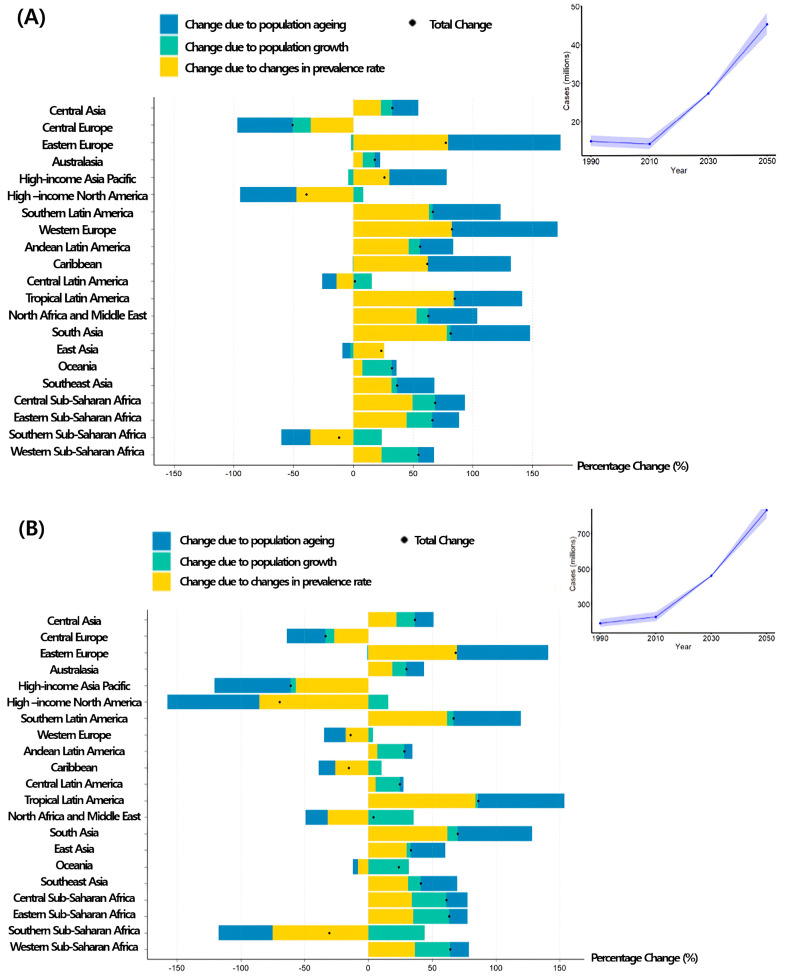
Decomposition of the projected change in the number of burn injury cases involving <20% (**A**) and ≥20% (**B**) total body surface area (TBSA) between 2020 and 2050.

By 2050, the number of severe burn cases is projected to reach 43.31 million (95% UI: 38.20–47.25 million), representing a 233.4% increase from 2020. Cases of mild burns are expected to increase to 570.74 million (95% UI: 533.15–646.40 million), reflecting a 142.5% increase from 2030 (Figure 3 and Table 2).

Regionally, Eastern Europe is expected to experience the most significant increase in severe burns, from 2.1 million cases in 2030 to 4.48 million in 2050 (113% increase). Meanwhile, Central America is projected to experience a decline owing to lower overall prevalence. In terms of mild burns, Sub-Saharan Africa is expected to experience a decline, with cases expected to decrease from 1.47 million in 2030 to 0.87 million in 2050.

These findings underscore regional disparities in the burden of burns and the disproportionate increase in severe burns compared with mild burns, highlighting the urgent need for targeted public health policies and prevention strategies to mitigate future burn-related morbidity and mortality.

## 4. Discussion

In this study, we estimated the global, regional, and national burden of burn injuries based on TBSA involvement and projected the changes in prevalence through 2050. Our findings indicate that while burn prevalence has generally declined in high-income countries (HICs), Eastern Europe is expected to experience the most notable increases, driven by industrialization, population aging, and a rise in occupational injuries. In 2021, approximately 240 million individuals worldwide were affected by burns, accounting for a substantial proportion of total YLDs [17]. By 2050, the global number of patients with burns is projected to reach 570 million, representing a 142.5% increase (95% UI: 118.1–147.3), with the largest increases anticipated in Eastern Europe and Central America. Among the key contributing factors, population aging is expected to be the primary driver of the rising burn burden in most regions by 2050 [18].

Although burns have a relatively low overall prevalence, they are a major health condition with considerable physical and psychological consequences [19]. In particular, severe burns involving more than 20% TBSA incur substantial treatment costs, requiring advanced surgical interventions, including skin grafting, prolonged intensive care, and extensive rehabilitation [20]. Additionally, continuous medical care for infection prevention, physical therapy, and skin regeneration treatments contributes to rising healthcare expenditures [21]. In LMICs, delays in medical intervention lead to a high risk of complications such as infections, sepsis, and organ failure, substantially reducing patient survival rates [22].

Burn wound healing is a complex, multi-phase process involving inflammation, tissue regeneration, and remodeling, which is severely disrupted in extensive burns and among older adults. Recent studies have shown that innovations in biomaterial-based dressings and hydrogel therapies can enhance outcomes by modulating immune responses and improving tissue regeneration [23]. However, these therapies remain largely inaccessible in low-resource settings, further exacerbating global disparities in burn recovery and long-term disability.

Burn injuries disproportionately affect the working-age population. Latin America and the Caribbean were the regions most affected among working-age adults [24]. In Eastern Europe, industrial accidents and traffic-related burns are major contributors to the burn burden, often resulting in substantial long-term work loss. For example, a study in the Czech Republic reported that approximately 25% of burn patients lost their jobs within six months post injury, with an average work absence exceeding 80 days [25]. In low-income countries (LICs) such as Cameroon, more than 30% of burn survivors lost their jobs within one year, with an average work loss of over 120 days, exacerbating household poverty and socioeconomic strain [26]. In the United States, there are burns approximately 4.8 million burn cases annually, with combined direct medical costs and productivity losses estimated at $7.6 billion per year [27].

Over the past three decades, HICs have experienced a decline in burn prevalence owing to advancements in healthcare systems, stricter fire safety regulations, and the implementation of burn prevention programs [28]. In contrast, LMICs continue to bear a disproportionately high burden of burns, with severe burns (TBSA > 20%) found to be more prevalent in regions lacking specialized burn care facilities [29]. Our projections for 2050 suggest that population growth, urbanization, and industrial expansion will contribute to a substantial rise in severe burns in Eastern Europe and Central America.

While milder burns (TBSA < 20%) have shown a growing trend in some high-income regions, such as Western Europe, this may be attributed to improvements in reporting systems and increased access to healthcare. These findings underscore the need for region-specific prevention and treatment strategies tailored to socioeconomic conditions [28].

In summary, our findings call for the development of region-specific burn prevention and treatment strategies that reflect the socioeconomic realities of each setting. Key policy recommendations include improving access to specialized burn care, enhancing community education on burn risks, and investing in cost-effective emergency response systems, particularly in low-resource settings.

Our findings align with previous studies that highlight disparities in burn burden, particularly in LMICs, where gaps in prevention and treatment remain key contributors to the high burn prevalence. However, this study expands on prior research by incorporating long-term projections, quantifying the anticipated increase in burden in vulnerable regions, and providing a more comprehensive global perspective rather than focusing on individual countries.

Multiple factors contribute to changes in burn prevalence. In HICs, advancements in fire safety policies, stricter occupational regulations, and improved emergency response systems have led to a decline in burn cases. Conversely, in LMICs, economic constraints, inadequate healthcare infrastructure, and delays in treatment continue to exacerbate burn-related mortality and disability burdens [8].

Additionally, climate change and increasing urbanization may influence burn injury patterns. Rapid economic growth in some nations has been linked to increased industrial accidents, underscoring the urgent need for stronger workplace safety regulations and improved emergency response frameworks. Furthermore, self-inflicted burns and interpersonal violence remain notable contributors to severe burn cases, emphasizing the importance of integrating mental health interventions into burn prevention strategies [30].

Since 1990, age-standardized burn prevalence and YLD rates have shown a moderate decline. While the precise causes remain elusive, this trend is likely driven by improved home and workplace safety measures, advancements in fire prevention technologies, and strengthened industrial safety policies [28]. However, severe burns continue to be a major source of disability and economic burden, necessitating a comprehensive global response.

To effectively address the projected burn burden, it is essential to critically assess the current limitations of prevention and treatment systems, particularly in LMICs. According to Gupta et al., an assessment of 458 hospitals in LMICs revealed that while more than 80% could provide initial burn management and basic resuscitation, only 35–38% had the capacity to deliver advanced burn care [31]. Specifically, the availability of skin grafting and management of complications was reported to be 35.6% and 37.9%, respectively. These findings suggest a significant treatment gap with severe burns (TBSA > 20%) increase. Furthermore, Botman et al. reported that in Tanzania, only 50% of burn patients reached a facility capable of surgical intervention within 24 h of injury, and in many institutions, treatment was limited to conservative management without surgical intervention [32]. Limited access to burn care in LMICs compromises timely and appropriate treatment, increasing the risk of poor outcomes. Strengthening the overall burn care system, including early management, surgical infrastructure, and workforce capacity, is therefore imperative to meet the anticipated burden.

Our findings indicate that prevention strategies should be income-specific. In HICs, policies should focus on improving access to specialized burn care and enhancing post-injury rehabilitation programs while LMICs need urgent infrastructure investments, emergency education, and low-cost treatment solutions. Implementing fire alarms, community prevention programs, and workplace safety protocols could significantly reduce burn incidence.

Recent studies have revealed that implementing fire detection systems and public safety campaigns can reduce residential fire-related burns by approximately 50%. Therefore, introducing low-cost fire alarms, community-based burn prevention programs, and strengthened workplace safety protocols should be prioritized in LMICs [33].

Our results indicate that severe burns (TBSA > 20%) become increasingly prevalent with age, peaking in individuals aged over 85 years. This finding is consistent with previous studies demonstrating that older patients experience greater functional decline, delayed wound healing, and higher risks of infection and complications [34]. Although an estimated 38.22% of severe burn-related YLDs were attributed to fire and heat, this proportion may not fully reflect the multifactorial nature of severe burn injuries. Contributing factors such as delayed treatment, socioeconomic vulnerability, and unsafe living or working environments often interact, amplifying both the incidence and severity of burns. Therefore, caution is warranted when interpreting single-cause attributions in burden analyses. Given the rising older population, tailored burn management strategies must be developed, incorporating longer hospital stays, infection prevention, and enhanced rehabilitation services. For example, the Centers for Disease Control and Prevention in the United States have implemented the STEADI program to prevent falls and burn-related injuries among older adults [17].

In addition to the elderly, females under 20 in low-resource settings are at elevated risk of severe burns due to early involvement in domestic tasks such as cooking and caregiving [35]. Exposure to open flames, boiling liquids, and traditional heating methods—often in poorly ventilated environments—combined with cultural factors like wearing loose-fitting garments, contributes to this vulnerability [36]. These factors help explain the higher burden observed in this demographic group. Accordingly, gender-sensitive prevention strategies are particularly critical in LMICs, where females under 20 are disproportionately exposed to domestic burn risks due to entrenched gender roles and unsafe household environments.

Unintentional injuries remain a major cause of burns worldwide, particularly those occurring at home, in workplaces, and during traffic accidents [37]. Children and older adults are at the highest risk of burns due to household fires, hot liquid exposure, and negligence, highlighting the need for improved safety education and household protective measures [38]. In rapidly industrializing countries, the incidence of chemical and electrical burns in workplaces is increasing, emphasizing the importance of strengthening occupational safety regulations. Traffic-related burns are also a crucial concern, particularly for motorcyclists and bicyclists, who are more vulnerable to severe burns in accidents. Addressing this issue requires enhanced road safety laws, improved protective gear mandates, and better emergency medical response systems [39].

Burn injuries resulting from self-harm and interpersonal violence are emerging as a critical public health issue worldwide, leading to severe physical and psychological consequences [19]. A rising trend in self-inflicted burns has been documented among adolescents and young adults, often associated with underlying mental health conditions [40]. These cases frequently involve scald and chemical burns, leaving long-term scars and functional impairments [41]. Meanwhile, burn injuries caused by interpersonal violence—such as domestic abuse, criminal acts, and armed conflicts—remain widespread, disproportionately affecting females and children. Beyond the immediate physical harm, these injuries frequently lead to chronic mental health issues and social stigmatization, considerably impacting the quality of life of survivors [42]. To reduce the burden of violence-related burns, stronger mental health support programs and expanded community-based prevention strategies are essential [43].

Our findings suggest that traffic accidents are among the major contributors to the global burn burden. According to the GBD study, traffic-related burns have been identified as a notable modifiable risk factor [42]. Motorcyclists, bicyclists, and pedestrians exhibit disproportionately high burn incidence rates, particularly in LMICs, where road safety measures remain inadequate. In certain countries, the implementation of mandatory helmet laws and enhanced road safety policies has contributed to a decline in burn-related injuries [44]. Nevertheless, traffic accident-related burns remain a serious issue in LICs, where insufficient workplace safety regulations and limited access to emergency medical services further exacerbate the burden [45].

Although this study offers valuable insights into the global burden of burns, its limitations should also be considered. First, variations in data reporting methods and classification systems across regions may introduce bias in prevalence estimates. For example, differences in case definitions, burn severity classifications, and data collection protocols can affect consistency and comparability between studies [46]. Moreover, hospital records and survey data from low- and middle-income countries (LMICs) often suffer from underreporting, especially among individuals who do not seek formal medical care [47]. This underreporting leads to an underestimate of the true prevalence of burn injuries in LMICs settings. Therefore, caution is required when interpreting and comparing burn prevalence data across different regions, and efforts to standardize data collection and classification systems are essential for improving the accuracy and reliability of global burn burden assessments. Second, the predictive model relies on current epidemiological trends and may not fully account for future advancements in medical technology or large-scale public health interventions. Furthermore, no sensitivity analyses were conducted to account for overlapping risk factors, such as the co-occurrence of self-harm and substance use, which may lead to under- or overestimation of specific causal attributions. Fourth, the projections did not account for climate-related factors such as wildfires and extreme heat, which may increasingly contribute to burn injuries in vulnerable regions. These limitations should be explored in future research.

Despite these limitations, this study has several notable strengths. First, it provides a comprehensive assessment of the global, regional, and national burden of burn injuries by integrating diverse data sources across multiple time points. Second, the study addresses an important gap in the literature by emphasizing the multifactorial nature of severe burns and highlighting the limitations of attributing burden solely to fire and heat. Third, the use of the DisMod-MR 2.1 hierarchical Bayesian meta-regression model strengthens the methodological rigor by improving the internal consistency of estimates for prevalence, incidence, mortality, and DALYs. Fourth, this is the first global analysis to stratify burn burden by total body surface area (TBSA) involvement, offering novel insights into severity-specific patterns across age groups and regions. Finally, by incorporating historical data from 1990 to 2021 and projecting trends through 2050, the study identifies high-risk regions and vulnerable populations, thereby informing targeted prevention strategies and long-term health policy planning.

## 5. Conclusions

In 2021, an estimated 250 million burn cases occurred globally, a number projected to exceed 600 million by 2050. Despite a decline in age-standardized rates, the absolute number of cases continues to rise owing to population growth and aging, with LMICs bearing the highest burden. Burns remain a major cause of disfigurement and reduced quality of life, underscoring the urgent need for targeted interventions in low- and middle-SDI regions.

## Figures and Tables

**Table 1:** Prevalence, YLDs, age-standardized rates of prevalence and YLDs per 100,000 population in 2020, and percentage change between 1990 and 2020 for burn injury involving <20% and ≥20% total body surface area (TBSA) globally by GBD regions and super-regions.

	TBSA	Number of Prevalent Cases	Age-Standardized Prevalence Rate per 100,000	Percentage Change in Age-Standardized Prevalence Rate from 1990 to 2021	Number of YLDs	Age-Standardized Rate of YLDs per 100,000	Percentage Change in Age-Standardized Rate of YLDs per 100,000 from 1990 to 2021
Global	Over 20	12,986,214 (11,964,280 to 14,293,813)	158.75 (146.05 to 174.66)	−0.54% (−0.56 to −0.51)	2998,693 (2058,722 to 3980,512)	158.75 (146.05 to 174.66)	−0.54% (−0.56 to −0.51)
	Under 20	235,335,949 (214,088,671 to 259,391,169)	2815.26 (2565.28 to 3104.57)	−0.31% (−0.32 to −0.29)	3733,288 (1988,493 to 6115,522)	2815.26 (2565.28 to 3104.57)	−0.31% (−0.32 to −0.29)
Central Europe, Eastern Europe, and Central Asia	Over 20	1,030,007 (967,433 to 1,106,280)	217.42 (203.41 to 233.73)	−0.67% (−0.68 to −0.65)	175,669 (123,428 to 233,759)	217.42 (203.41 to 233.73)	−0.67% (−0.68 to −0.65)
	Under 20	30,141,765 (27,408,292 to 33,035,656)	5829.6 (5286.44 to 6417.65)	−0.3% (−0.31 to −0.29)	475,102 (249,932 to 797,220)	5829.6 (5286.44 to 6417.65)	−0.3% (−0.31 to −0.29)
Central Asia	Over 20	324,419 (300,165 to 348,576)	326.57 (302.05 to 351.04)	−0.55% (−0.57 to −0.53)	70,193 (48,582 to 92,568)	326.57 (302.05 to 351.04)	−0.55% (−0.57 to −0.53)
	Under 20	5,082,101 (4,600,690 to 5,623,153)	5309.38 (4811.41 to 5868.86)	−0.29% (−0.31 to −0.28)	81,639 (43,018 to 135,660)	5309.38 (4811.41 to 5868.86)	−0.29% (−0.31 to −0.28)
Central Europe	Over 20	221,217 (206,774 to 239,432)	159.09 (148.31 to 172.82)	−0.79% (−0.82 to −0.77)	30,918 (21,748 to 41,820)	159.09 (148.31 to 172.82)	−0.79% (−0.82 to −0.77)
	Under 20	9,035,437 (8,192,377 to 9,979,794)	5996.47 (5467.77 to 6655.23)	−0.3% (−0.31 to −0.29)	141,997 (74,630 to 237,957)	5996.47 (5467.77 to 6655.23)	−0.3% (−0.31 to −0.29)
Eastern Europe	Over 20	484,371 (455,108 to 519,086)	193.64 (181.95 to 208.47)	−0.7% (−0.72 to −0.68)	74,558 (52,442 to 99,598)	193.64 (181.95 to 208.47)	−0.7% (−0.72 to −0.68)
	Under 20	16,024,228 (14,562,282 to 17,623,600)	5919.18 (5383.66 to 6515.17)	−0.29% (−0.31 to −0.28)	251,466 (132,284 to 425,157)	5919.18 (5383.66 to 6515.17)	−0.29% (−0.31 to −0.28)
High-income	Over 20	247,807 (229,962 to 267,821)	18.81 (17.37 to 20.33)	−0.53% (−0.56 to −0.5)	34,447 (24,149 to 46,929)	18.81 (17.37 to 20.33)	−0.53% (−0.56 to −0.5)
	Under 20	53,148,453 (47,020,573 to 60,451,241)	3749.41 (3321.14 to 4283.84)	−0.3% (−0.31 to −0.29)	834,071 (444,657 to 1,411,194)	3749.41 (3321.14 to 4283.84)	−0.3% (−0.31 to −0.29)
Australasia	Over 20	8,480 (7,786 to 9,265)	23.4 (21.41 to 25.72)	−0.22% (−0.34 to −0.1)	1123 (756 to 1586)	23.4 (21.41 to 25.72)	−0.22% (−0.34 to −0.1)
	Under 20	2,006,433 (1,780,284 to 2,291,976)	5309.87 (4694.81 to 6083.1)	−0.22% (−0.24 to −0.2)	31,642 (16,797 to 53,621)	5309.87 (4694.81 to 6083.1)	−0.22% (−0.24 to −0.2)
High-income Asia Pacific	Over 20	44,284 (40,605 to 47,991)	17.67 (16.19 to 19.34)	−0.65% (−0.69 to −0.61)	5,810 (3,919 to 8,054)	17.67 (16.19 to 19.34)	−0.65% (−0.69 to −0.61)
	Under 20	10,481,157 (9,166,205 to 12,042,913)	3964.64 (3471.22 to 4591.86)	−0.32% (−0.33 to −0.3)	164,605 (87,258 to 277,958)	3964.64 (3471.22 to 4591.86)	−0.32% (−0.33 to −0.3)
High-income North America	Over 20	65,062 (60,102 to 70,093)	13.99 (12.91 to 15.12)	−0.4% (−0.44 to −0.36)	8,534 (5,844 to 11,796)	13.99 (12.91 to 15.12)	−0.4% (−0.44 to −0.36)
	Under 20	14,867,755 (13,078,151 to 16,948,823)	3103.54 (2724.96 to 3544.37)	−0.39% (−0.4 to −0.38)	231,492 (124,291 to 389,680)	3103.54 (2724.96 to 3544.37)	−0.39% (−0.4 to −0.38)
Southern Latin America	Over 20	44,367 (39,945 to 49,377)	61.22 (55.04 to 68.5)	−0.66% (−0.7 to −0.61)	7,739 (5,302 to 10,416)	61.22 (55.04 to 68.5)	−0.66% (−0.7 to −0.61)
	Under 20	5,949,381 (5,281,328 to 6,794,015)	7836.51 (6939.49 to 8972.91)	−0.22% (−0.24 to −0.2)	94,644 (49,835 to 159,569)	7836.51 (6939.49 to 8972.91)	−0.22% (−0.24 to −0.2)
Western Europe	Over 20	85,615 (78,967 to 93,047)	16.01 (14.76 to 17.46)	−0.46% (−0.5 to −0.42)	11,240 (7842 to 15,631)	16.01 (14.76 to 17.46)	−0.46% (−0.5 to −0.42)
	Under 20	19,843,727 (17,625,663 to 22,510,607)	3485.95 (3102.34 to 3968.37)	−0.27% (−0.28 to −0.26)	311,688 (165,349 to 528,911)	3485.95 (3102.34 to 3968.37)	−0.27% (−0.28 to −0.26)
Latin America and the Caribbean	Over 20	2,155,188 (1,958,476 to 2,356,944)	346.73 (314.8 to 379.82)	−0.62% (−0.64 to −0.59)	469,785 (330,106 to 634,935)	346.73 (314.8 to 379.82)	−0.62% (−0.64 to −0.59)
	Under 20	27,180,070 (24,587,197 to 30,673,580)	4331.94 (3913.66 to 4889.19)	−0.33% (−0.34 to −0.31)	432,444 (229,289 to 716,585)	4331.94 (3913.66 to 4889.19)	−0.33% (−0.34 to −0.31)
Andean Latin America	Over 20	185,479 (168,442 to 205,096)	276.57 (251.54 to 305.69)	−0.68% (−0.7 to −0.66)	38,895 (27,092 to 52,279)	276.57 (251.54 to 305.69)	−0.68% (−0.7 to −0.66)
	Under 20	2,625,401 (2,369,720 to 2,959,323)	3996.91 (3609.25 to 4498.12)	−0.26% (−0.28 to −0.24)	42,054 (22,111 to 70,426)	3996.91 (3609.25 to 4498.12)	−0.26% (−0.28 to −0.24)
Caribbean	Over 20	299,796 (248,537 to 379,847)	626.94 (516.55 to 802.54)	0.08% (−0.14 to 0.44)	83,401 (55,712 to 123,215)	626.94 (516.55 to 802.54)	0.08% (−0.14 to 0.44)
	Under 20	2,994,857 (2,651,723 to 3,446,551)	5976.04 (5284.74 to 6900.3)	0.01% (−0.05 to 0.11)	47,545 (25,884 to 78,984)	5976.04 (5284.74 to 6900.3)	0.01% (−0.05 to 0.11)
Central Latin America	Over 20	1,069,834 (959,276 to 1,199,999)	407.23 (365.35 to 456.89)	−0.69% (−0.7 to −0.67)	223,457 (156,659 to 300,616)	407.23 (365.35 to 456.89)	−0.69% (−0.7 to −0.67)
	Under 20	12,445,153 (11,001,937 to 14,596,375)	4755.14 (4203.04 to 5578.74)	−0.36% (−0.37 to −0.34)	198,675 (103,686 to 329,029)	4755.14 (4203.04 to 5578.74)	−0.36% (−0.37 to −0.34)
Tropical Latin America	Over 20	600,078 (561,238 to 643,957)	241.56 (225.81 to 259.38)	−0.63% (−0.65 to −0.61)	124,032 (86,462 to 163,438)	241.56 (225.81 to 259.38)	−0.63% (−0.65 to −0.61)
	Under 20	9,114,659 (8,310,295 to 9,998,960)	3615.21 (3297.93 to 3967.26)	−0.37% (−0.38 to −0.36)	144,170 (77,651 to 242,786)	3615.21 (3297.93 to 3967.26)	−0.37% (−0.38 to −0.36)
North Africa and the Middle East	Over 20	1,153,602 (959,851 to 1,591,012)	181.53 (151.11 to 250.28)	−0.63% (−0.67 to −0.57)	242,801 (160,980 to 360,674)	181.53 (151.11 to 250.28)	−0.63% (−0.67 to −0.57)
	Under 20	17,658,442 (15,668,136 to 20,247,212)	2924.02 (2600.11 to 3338.97)	−0.29% (−0.32 to −0.24)	282,018 (158,044 to 464,259)	2924.02 (2600.11 to 3338.97)	−0.29% (−0.32 to −0.24)
South Asia	Over 20	3,221,488 (2,977,649 to 3,513,470)	171.08 (158.45 to 186.01)	−0.47% (−0.5 to −0.44)	813,814 (560,360 to 1,086,685)	171.08 (158.45 to 186.01)	−0.47% (−0.5 to −0.44)
	Under 20	32,901,256 (29,594,944 to 36,317,130)	1844.24 (1665.64 to 2026.5)	−0.21% (−0.23 to −0.17)	521,926 (287,884 to 869,041)	1844.24 (1665.64 to 2026.5)	−0.21% (−0.23 to −0.17)
Southeast Asia, East Asia, and Oceania	Over 20	2,734,119 (2,529,183 to 2,988,281)	112.1 (103.2 to 123.97)	−0.69% (−0.72 to −0.66)	545,174 (387,856 to 730,870)	112.1 (103.2 to 123.97)	−0.69% (−0.72 to −0.66)
	Under 20	54,230,940 (49,291,833 to 59,781,048)	2116.24 (1917.82 to 2337.6)	−0.14% (−0.16 to −0.1)	862,106 (458,703 to 1,427,636)	2116.24 (1917.82 to 2337.6)	−0.14% (−0.16 to −0.1)
East Asia	Over 20	1,257,609 (1,187,835 to 1,340,643)	69.54 (65.65 to 74.43)	−0.85% (−0.87 to −0.83)	188,256 (131,597 to 257,190)	69.54 (65.65 to 74.43)	−0.85% (−0.87 to −0.83)
	Under 20	35,651,573 (32,322,563 to 39,460,461)	1935.22 (1750.03 to 2142.56)	−0.15% (−0.17 to −0.12)	565,634 (299,152 to 951,251)	1935.22 (1750.03 to 2142.56)	−0.15% (−0.17 to −0.12)
Oceania	Over 20	48,137 (43,675 to 53,151)	361.66 (330.28 to 397.72)	0.12% (0.04 to 0.21)	14,203 (9802 to 19,295)	361.66 (330.28 to 397.72)	0.12% (0.04 to 0.21)
	Under 20	379,711 (338,199 to 432,344)	3235.43 (2894.44 to 3629.03)	0.11% (0.06 to 0.2)	6099 (3393 to 10,098)	3235.43 (2894.44 to 3629.03)	0.11% (0.06 to 0.2)
Southeast Asia	Over 20	1,428,372 (1,280,936 to 1,639,285)	191.33 (171.5 to 220.52)	−0.48% (−0.53 to −0.41)	342,715 (240,757 to 466,829)	191.33 (171.5 to 220.52)	−0.48% (−0.53 to −0.41)
	Under 20	18,199,656 (16,355,484 to 20,232,577)	2489.55 (2240.38 to 2765.48)	−0.18% (−0.21 to −0.12)	290,373 (161,304 to 473,800)	2489.55 (2240.38 to 2765.48)	−0.18% (−0.21 to −0.12)
Sub-Saharan Africa	Over 20	2,444,003 (2,180,070 to 2,804,442)	244.68 (219.52 to 282.98)	−0.36% (−0.38 to −0.33)	717,003 (482,999 to 961,282)	244.68 (219.52 to 282.98)	−0.36% (−0.38 to −0.33)
	Under 20	20,075,024 (18,070,533 to 22,545,280)	2384.35 (2148.05 to 2674.4)	−0.22% (−0.23 to −0.19)	325,621 (175,932 to 543,005)	2384.35 (2148.05 to 2674.4)	−0.22% (−0.23 to −0.19)
Central Sub-Saharan Africa	Over 20	277,087 (243,127 to 337,253)	233.71 (204.41 to 290.73)	−0.31% (−0.37 to −0.24)	84,061 (57,266 to 113,930)	233.71 (204.41 to 290.73)	−0.31% (−0.37 to −0.24)
	Under 20	2,027,773 (1,804,737 to 2,331,587)	2025.48 (1806.75 to 2310.22)	−0.17% (−0.21 to −0.09)	32,729 (18,309 to 54,503)	2025.48 (1806.75 to 2310.22)	−0.17% (−0.21 to −0.09)
Eastern Sub-Saharan Africa	Over 20	1,070,985 (932,224 to 1,288,720)	287.21 (249.77 to 353.16)	−0.35% (−0.37 to −0.32)	320,864 (213,990 to 437,477)	287.21 (249.77 to 353.16)	−0.35% (−0.37 to −0.32)
	Under 20	8,231,034 (7,308,373 to 9,420,734)	2633.93 (2342.65 to 3007.61)	−0.23% (−0.24 to −0.2)	133,660 (72,737 to 224,121)	2633.93 (2342.65 to 3007.61)	−0.23% (−0.24 to −0.2)
Southern Sub-Saharan Africa	Over 20	179,570 (166,694 to 193,336)	216.55 (201.54 to 233.08)	−0.55% (−0.57 to −0.54)	46,247 (31,906 to 60,620)	216.55 (201.54 to 233.08)	−0.55% (−0.57 to −0.54)
	Under 20	2,200,763 (1,992,089 to 2,457,356)	2924.62 (2645.94 to 3266.93)	−0.32% (−0.33 to −0.31)	35,011 (18,644 to 59,054)	2924.62 (2645.94 to 3266.93)	−0.32% (−0.33 to −0.31)
Western Sub-Saharan Africa	Over 20	916,360 (833,392 to 1,008,105)	216.34 (198.77 to 237.09)	−0.31% (−0.34 to −0.28)	265,832 (179,755 to 355,702)	216.34 (198.77 to 237.09)	−0.31% (−0.34 to −0.28)
	Under 20	7,615,454 (6,922,026 to 8,428,159)	2122.19 (1930.91 to 2345.05)	−0.15% (−0.16 to −0.12)	124,221 (66,325 to 205,240)	2122.19 (1930.91 to 2345.05)	−0.15% (−0.16 to −0.12)

Data in parentheses are 95% uncertainty intervals. Region and super-region numbers do not sum to the global prevalence due to rounding. YLDs, years lived with disability.

**Table 2:** Age-standardized prevalence and cases of burn injury involving <20% and ≥20% total body surface area (TBSA) projections up to 2030, 2040, and 2050, globally and by region, with male and female sexes combined.

	TBSA	Age-Standardized Prevalence			Cases (Millions)		
		2030	2040	2050	2030	2040	2050
Global	Over 20	0.33% (0.3–0.36)	0.39% (0.34–0.42)	0.45% (0.4–0.49)	28.32 (25.52–30.84)	35.43 (31.58–38.61)	43.31 (38.2–47.25)
	Under 20	4.71% (4.36–5.27)	5.31% (4.93–5.98)	5.98% (5.58–6.77)	406.38 (375.37–454.58)	487.32 (452.81–548.68)	570.74 (533.15–646.4)
Andean Latin America	Over 20	0.45% (0.41–0.51)	0.46% (0.42–0.52)	0.46% (0.43–0.54)	0.33 (0.31–0.38)	0.38 (0.35–0.43)	0.42 (0.39–0.48)
	Under 20	4.49% (3.94–4.94)	4.38% (3.79–4.78)	4.28% (3.65–4.63)	3.34 (2.93–3.67)	3.62 (3.13–3.95)	3.83 (3.27–4.14)
Australasia	Over 20	0.03% (0.03–0.03)	0.03% (0.03–0.03)	0.03% (0.02–0.03)	0.01 (0.01–0.01)	0.01 (0.01–0.01)	0.01 (0.01–0.01)
	Under 20	7.1% (6.57–8.41)	7.36% (6.95–8.83)	7.63% (7.34–9.27)	2.34 (2.16–2.77)	2.64 (2.49–3.16)	2.9 (2.79–3.52)
Caribbean	Over 20	1.13% (0.99–1.38)	1.49% (1.31–1.84)	1.96% (1.73–2.45)	0.56 (0.49–0.68)	0.75 (0.66–0.93)	0.97 (0.86–1.21)
	Under 20	3.89% (3.37–4.63)	3.21% (2.75–3.88)	2.64% (2.23–3.24)	1.92 (1.67–2.29)	1.61 (1.38–1.95)	1.31 (1.11–1.6)
Central Asia	Over 20	0.52% (0.48–0.54)	0.53% (0.5–0.54)	0.55% (0.51–0.55)	0.54 (0.5–0.56)	0.6 (0.56–0.62)	0.67 (0.62–0.67)
	Under 20	7.3% (6.67–7.94)	7.53% (6.89–8.14)	7.78% (7.13–8.35)	7.64 (6.98–8.31)	8.56 (7.83–9.25)	9.45 (8.66–10.15)
Central Europe	Over 20	0.12% (0.12–0.13)	0.08% (0.08–0.09)	0.06% (0.06–0.06)	0.13 (0.13–0.14)	0.08 (0.08–0.09)	0.05 (0.05–0.06)
	Under 20	4.78% (4.3–5.47)	3.96% (3.55–4.59)	3.28% (2.93–3.86)	5.22 (4.7–5.97)	4.07 (3.65–4.72)	3.12 (2.78–3.66)
Central Latin America	Over 20	0.44% (0.38–0.47)	0.38% (0.33–0.4)	0.33% (0.28–0.34)	1.3 (1.13–1.4)	1.2 (1.04–1.27)	1.08 (0.93–1.13)
	Under 20	5.57% (4.81–6.38)	5.42% (4.64–6.15)	5.29% (4.49–5.92)	16.44 (14.22–18.84)	17.21 (14.71–19.5)	17.49 (14.83–19.59)
Central Sub-Saharan Africa	Over 20	0.37% (0.27–0.68)	0.41% (0.28–0.9)	0.46% (0.29–1.18)	0.62 (0.46–1.16)	0.86 (0.59–1.88)	1.13 (0.72–2.92)
	Under 20	2.71% (2.3–3.29)	2.89% (2.39–3.62)	3.08% (2.49–3.96)	4.62 (3.91–5.61)	6.05 (5.01–7.58)	7.59 (6.15–9.78)
East Asia	Over 20	0.07% (0.06–0.08)	0.05% (0.05–0.06)	0.04% (0.03–0.04)	1.05 (0.95–1.13)	0.76 (0.68–0.81)	0.53 (0.47–0.57)
	Under 20	2.52% (2.39–2.69)	2.69% (2.58–2.84)	2.87% (2.8–2.99)	37.66 (35.67–40.25)	38.58 (37.07–40.7)	38.45 (37.51–40.08)
Eastern Europe	Over 20	1.04% (1–1.1)	1.6% (1.54–1.68)	2.45% (2.38–2.54)	2.1 (2.01–2.21)	3.08 (2.97–3.22)	4.48 (4.35–4.65)
	Under 20	16.02% (14.72–17.55)	20.61% (19.04–22.53)	26.17% (24.25–28.41)	32.24 (29.63–35.33)	39.62 (36.6–43.32)	47.82 (44.31–51.91)
Eastern Sub-Saharan Africa	Over 20	0.38% (0.28–0.75)	0.39% (0.28–0.93)	0.41% (0.27–1.15)	2.09 (1.55–4.14)	2.69 (1.89–6.36)	3.3 (2.19–9.28)
	Under 20	3.63% (3.15–4.19)	3.87% (3.32–4.49)	4.13% (3.5–4.81)	20.15 (17.48–23.26)	26.53 (22.75–30.76)	33.25 (28.21–38.73)
High-income Asia Pacific	Over 20	0.04% (0.04–0.05)	0.05% (0.05–0.05)	0.07% (0.06–0.07)	0.08 (0.07–0.08)	0.09 (0.08–0.1)	0.11 (0.1–0.11)
	Under 20	2.31% (2.08–2.64)	1.63% (1.48–1.85)	1.15% (1.05–1.29)	4.22 (3.79–4.82)	2.83 (2.57–3.21)	1.85 (1.69–2.08)
High-income North America	Over 20	0.01% (0.01–0.01)	0% (0–0)	0% (0–0)	0.02 (0.02–0.02)	0.01 (0.01–0.01)	0.01 (0.01–0.01)
	Under 20	1.22% (1.07–1.42)	0.76% (0.66–0.88)	0.47% (0.41–0.55)	4.75 (4.16–5.51)	3.05 (2.66–3.55)	1.9 (1.66–2.22)
North Africa and Middle East	Over 20	0.08% (0.06–0.13)	0.05% (0.03–0.08)	0.03% (0.02–0.06)	0.6 (0.44–0.93)	0.42 (0.28–0.69)	0.28 (0.18–0.5)
	Under 20	2.33% (2.03–3.18)	1.95% (1.68–2.9)	1.63% (1.39–2.63)	17.05 (14.85–23.31)	16.04 (13.83–23.84)	14.67 (12.5–23.68)
Oceania	Over 20	0.3% (0.3–0.31)	0.28% (0.28–0.29)	0.27% (0.27–0.26)	0.05 (0.05–0.05)	0.06 (0.05–0.06)	0.06 (0.06–0.06)
	Under 20	2.52% (2.26–2.76)	2.27% (2.04–2.46)	2.04% (1.84–2.2)	0.41 (0.37–0.45)	0.44 (0.4–0.48)	0.46 (0.42–0.5)
South Asia	Over 20	0.47% (0.42–0.51)	0.63% (0.55–0.69)	0.85% (0.72–0.93)	9.39 (8.31–10.19)	13.22 (11.48–14.37)	18.02 (15.35–19.62)
	Under 20	3.78% (3.41–4.2)	4.71% (4.25–5.26)	5.86% (5.29–6.56)	75.45 (68.14–83.85)	98.45 (88.85–109.89)	124.13 (112.03–139)
Southeast Asia	Over 20	0.32% (0.25–0.38)	0.36% (0.26–0.43)	0.4% (0.27–0.49)	2.37 (1.85–2.82)	2.76 (2.03–3.34)	3.11 (2.15–3.84)
	Under 20	4.13% (3.51–4.76)	4.79% (3.96–5.61)	5.55% (4.47–6.6)	30.44 (25.83–35.08)	37.05 (30.6–43.35)	43.65 (35.14–51.91)
Southern Latin America	Over 20	0.17% (0.14–0.21)	0.22% (0.18–0.28)	0.28% (0.22–0.37)	0.12 (0.1–0.15)	0.17 (0.13–0.21)	0.22 (0.17–0.29)
	Under 20	18.2% (16.77–22.03)	23.23% (21.73–28.51)	29.17% (27.67–36)	13.17 (12.13–15.94)	17.6 (16.46–21.6)	22.66 (21.49–27.96)
Southern Sub–Saharan Africa	Over 20	0.27% (0.24–0.29)	0.25% (0.23–0.28)	0.25% (0.22–0.27)	0.25 (0.23–0.27)	0.26 (0.24–0.29)	0.27 (0.25–0.3)
	Under 20	1.59% (1.44–1.78)	1.11% (1.01–1.25)	0.78% (0.71–0.88)	1.47 (1.33–1.65)	1.15 (1.04–1.29)	0.87 (0.79–0.98)
Tropical Latin America	Over 20	0.95% (0.87–1.02)	1.37% (1.25–1.49)	1.99% (1.8–2.16)	2.25 (2.07–2.43)	3.35 (3.06–3.63)	4.84 (4.39–5.25)
	Under 20	14.75% (13.83–16.55)	22.07% (20.94–24.79)	31.68% (30.42–35.39)	35.08 (32.88–39.36)	53.83 (51.08–60.48)	77.09 (74.03–86.13)
Western Europe	Over 20	0.07% (0.07–0.08)	0.12% (0.12–0.12)	0.21% (0.21–0.2)	0.33 (0.31–0.33)	0.56 (0.54–0.55)	0.94 (0.91–0.9)
	Under 20	3.32% (2.94–3.83)	2.96% (2.62–3.44)	2.64% (2.34–3.08)	14.78 (13.09–17.04)	13.27 (11.74–15.39)	11.69 (10.33–13.64)
Western Sub–Saharan Africa	Over 20	0.22% (0.19–0.25)	0.21% (0.18–0.24)	0.2% (0.17–0.23)	1.38 (1.22–1.55)	1.68 (1.47–1.91)	1.97 (1.7–2.25)
	Under 20	3% (2.7–3.34)	3.3% (2.95–3.69)	3.63% (3.23–4.08)	18.84 (16.94–20.96)	26.53 (23.71–29.64)	35.71 (31.74–40.08)

Data in parentheses are 95% uncertainty intervals. Regional numbers do not sum to the global prevalence due to rounding.

## Data Availability

The datasets generated and/or analyzed during the current study are available at https://www.healthdata.org/research-analysis/gbd (accessed on 10 February 2025).

## Abbreviations

The following abbreviations are used in this manuscript:

DALY	Disability-adjusted life years
GBD	Global Burden of Disease
HIC	High-income countries
LIC	Low-income countries
LMIC	Low- and middle-income countries
SDI	Socio-demographic index
TBSA	Total body surface area
UI	Uncertainty intervals
YLD	Years lived with disability
